# Supplementary figures and images for: Natural language processing for analyzing online customer reviews: a survey, taxonomy, and open research challenges
Source: PeerJ Comput Sci. 2024 Jul 19;10:e2203. doi: 10.7717/peerj-cs.2203 (PMC11323031; doi:10.7717/peerj-cs.2203)

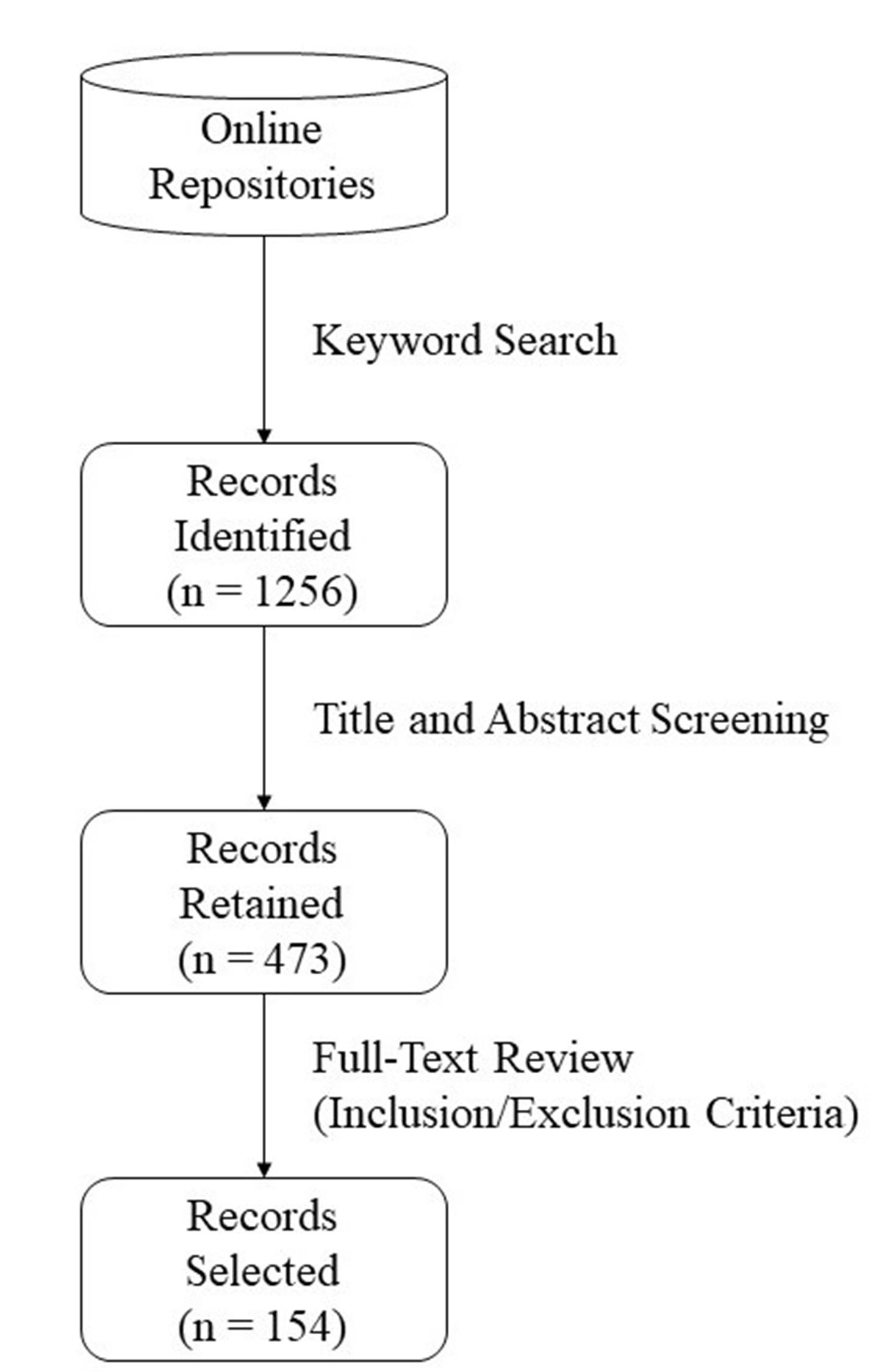

Supplement: Supplemental Information 1 [file peerj-cs-10-2203-s001.jpg]

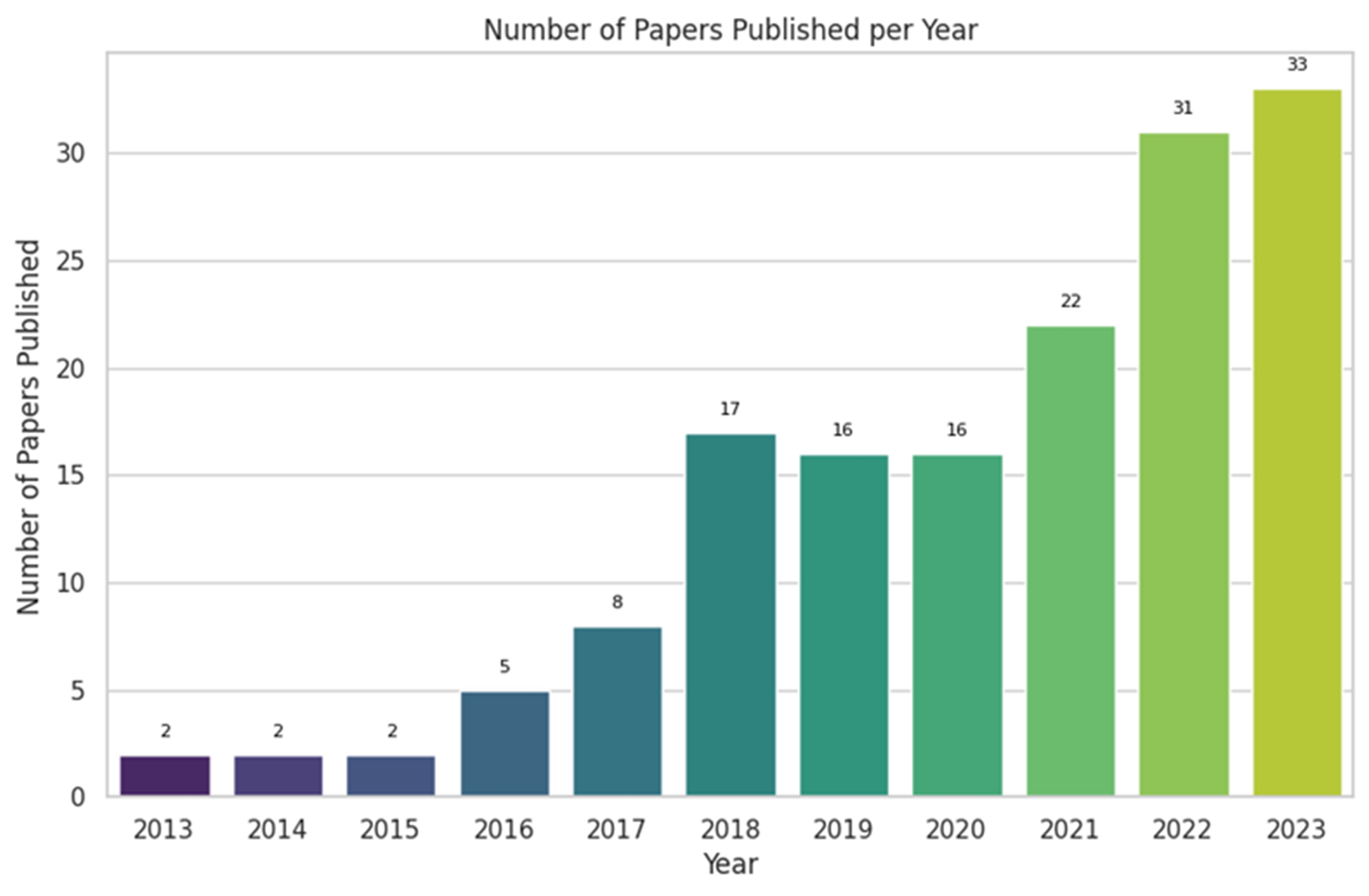

Supplement: Supplemental Information 2 [file peerj-cs-10-2203-s002.jpg]
