# Supplementary material for: Natural language processing for analyzing online customer reviews: a survey, taxonomy, and open research challenges
Source: PeerJ Comput Sci. 2024 Jul 19;10:e2203. doi: 10.7717/peerj-cs.2203 (PMC11323031; doi:10.7717/peerj-cs.2203)
Supplement: Supplemental Information 3 [file peerj-cs-10-2203-s003.docx]

Supplemental Table S1. Inclusion and Exclusion Criteria.

| Inclusion Criteria | Exclusion Criteria |
| --- | --- |
| Papers directly address topics related to online reviews, customer feedback, or NLP within the context of electronic commerce | Papers not focusing on the intersection of NLP and online reviews within the context of electronic commerce are excluded |
| Papers published from 2013 to 2023 | Papers published before 2013 are excluded |
| Papers written in English are included | Papers not written in English are excluded |
| Papers eligible for inclusion include conference papers, articles, and chapters | Papers that are not conference papers, articles, or chapters are excluded |
