# Supplementary material for: Natural language processing for analyzing online customer reviews: a survey, taxonomy, and open research challenges
Source: PeerJ Comput Sci. 2024 Jul 19;10:e2203. doi: 10.7717/peerj-cs.2203 (PMC11323031; doi:10.7717/peerj-cs.2203)
Supplement: Supplemental Information 4 [file peerj-cs-10-2203-s004.docx]

Supplemental Table S2. Summary of literature on sentiment analysis and opinion mining.

| Ref. | Year | Dataset | Description |
| --- | --- | --- | --- |
| (Marrese-Taylor et al., 2014) | 2014 | TripAdvisor | Introduced enhanced opinion mining for tourism, outperformed existing models in sentiment classification in Chile. |
| (Singla et al., 2017) | 2017 | Amazon, Flipkart | Utilized Sentiment Analysis on mobile reviews for consumer decision-making. |
| (Fazzolari et al., 2017) | 2017 | TripAdvisor | Employed AI tools revealing mismatches between sentiment and scores in hotel reviews. |
| (Li & Yang, 2017) | 2017 | TripAdvisor | Presented Chinese sentiment mining outperforming existing models in reviews. |
| (Magesh & Swarnalatha, 2017) | 2017 | Amazon | Detailed a methodology using NLP and machine learning for sentiment classification in book reviews. |
| (Dufourq & Bassett, 2017) | 2017 | Amazon | Presented two Genetic Algorithms for automated text sentiment analysis, outperforming existing methods. |
| (Jain et al., 2018) | 2018 | IMDb, Amazon | Investigated the application of deep neural networks, showcasing superior performance compared to traditional methods. |
| (Gawade & Parthiban, 2018) | 2018 | Amazon | Highlighted the importance of opinion mining in e-commerce, aiming to develop a machine for sentiment analysis. |
| (Aryo Prakoso et al., 2018) | 2018 | Amazon | Utilized NLP and a UCI machine learning dataset to assess Amazon customer reviews. |
| (Panthati et al., 2018) | 2018 | Amazon | Introduced a sentiment analysis approach for product reviews, utilizing deep learning with word2vec. |
| (Wang et al., 2018) | 2018 | Amazon | Introduced a cost-effective approach to product design through effective engineering. |
| (Angioni et al., 2018) | 2018 | Yelp | Presented a new method to evaluate Opinion Mining system performance by incorporating user preferences. |
| (Karthikayini & Srinath, 2018) | 2018 | Amazon | Addressed the challenges of opinion mining in handling diverse online user data. |
| (Bansal & Srivastava, 2019) | 2019 | TripAdvisor, Amazon | Proposed HABSC, a novel method leveraging syntactic features, implicit word relations, and domain-specific knowledge. |
| (Nakayama & Wan, 2019) | 2019 | Yelp | Explored the influence of ethnic culture on customer reviews in social commerce. |
| (Chauhan & Sehgal, 2019) | 2019 | Large Mobile Review Dataset | Investigated sentiment analysis in NLP. |
| (Jabbar et al., 2019) | 2019 | Amazon | Utilized in machine learning to perform sentiment analysis on E-commerce product reviews. |
| (Pathuri et al., 2019) | 2019 | Amazon | Explored sentiment analysis in NLP's context, emphasizing its pivotal role in Business Analytics. |
| (Dharaiya et al., 2020) | 2020 | Amazon | Proposed a model to overcome the limitations of traditional online product analysis. |
| (Jamadi Khiabani et al., 2020) | 2020 | TripAdvisor, CitySearch | Presented a novel sentiment analysis method, utilizing a two-point structure to enhance the Dempster–Shafer algorithm. |
| (Khatun et al., 2020) | 2020 | Amazon | Explored sentiment analysis in e-commerce, specifically on Amazon, to assess book and author quality. |
| (D. P. Chatterjee et al., 2021) | 2021 | Check | Compared the effectiveness of LSTM, random forest, SVM, and XGBoost in both binary and multiclass scenarios. |
| (Bhamare & Prabhu, 2021) | 2021 | SemEval, Yelp, Kaggle datasets | Proposed two methods for aspect extraction in Aspect-Based Sentiment Analysis from unstructured social media reviews. |
| (Mukherjee et al., 2021) | 2021 | Amazon | Introduced a novel sentiment analysis approach with a customized negation marking algorithm. |
| (Gupta et al., 2021) | 2021 | Amazon | Introduced a sentiment analysis mechanism employing machine learning algorithms. |
| (Khanam & Sharma, 2021) | 2021 | - | Investigated sentiment analysis on WWW content, utilizing a lexicon-based method and logistic regression in machine learning. |
| (I. Chatterjee et al., 2021) | 2021 | Amazon | Investigated sentiment analysis and outlier detection in Amazon customer reviews. |
| (Urkude et al., 2021) | 2021 | Amazon | Focused on sentiment analysis of Amazon electronics product reviews. |
| (Chen & Yao, 2021) | 2021 | Yelp, IMDb, Amazon | Introduced a sentiment analysis model addressing challenges in data pre-processing and classification uncertainty. |
| (Hawlader et al., 2021) | 2021 | Amazon | Investigated the role of machine learning, employing diverse classifiers and preprocessing techniques. |
| (Geetha & Karthika Renuka, 2021) | 2021 | Amazon | Improved sentiment analysis of E-commerce reviews by introducing the BERT Base Uncased model. |
| (Sinnasamy & Sjaif, 2022) | 2022 | Amazon | Employed NLP, utilizing term-based methods and N-grams. |
| (Maurya & Pratap, 2022) | 2022 | Amazon | Explored the importance of online product reviews, employing an Ensemble Classifier. |
| (Naureen et al., 2022) | 2022 | Amazon | Employed SVM, random forest, and naive bayes algorithms to enhance sentiment analysis for Amazon products. |
| (Chu et al., 2022) | 2022 | TripAdvisor | Outlined a novel aspect-based sentiment analysis model, leveraging BERT, to extract sentiment and aspect-category information. |
| (Nasfi & Bouguila, 2022) | 2022 | Amazon, IMDb | Introduced a hybrid generative-discriminative approach using Fisher kernels and hidden Markov models. |
| (Yarkareddy et al., 2022) | 2022 | Amazon | Investigated the role of online reviews in the digitized e-commerce landscape, utilizing machine learning algorithms. |
| (Roccabruna et al., 2022) | 2022 | Twitter, YouTube, Facebook, Amazon, TripAdvisor | Investigated sentiment analysis on Italian corpora using BERT-based models. |
| (Juyal, 2022) | 2022 | Amazon | Proposed a hybrid approach, leveraging NLP, machine learning, and Deep Learning. |
| (Uma et al., 2022) | 2022 | Amazon | Employed SVM and CNN Models for Customer Review Sentiment Analysis. |
| (Kamalesh & Vijayalakshmi, 2022) | 2022 | Amazon | Outlined a machine learning-based sentiment evaluation model for e-commerce shopper reviews. |
| (Singh et al., 2022) | 2022 | Amazon | Utilized NLP to automate the analysis of product reviews on platforms like Amazon. |
| (Yang et al., 2022) | 2022 | Yelp, Zappos | Investigated the impact of NLP models on consumer reviews from Yelp and Zappos. |
| (Gupta et al., 2022) | 2022 | Amazon | Introduced a model designed for the food industry, utilizing NLP techniques and machine learning classification algorithms. |
| (Hariharan et al., 2023) | 2023 | - | Introduced a Bayesian-network framework for automated sentence-level sentiment analysis on e-commerce websites. |
| (Maheswari & Dhenakaran, 2023) | 2023 | Amazon | Pioneered a method for effectively identifying sarcastic opinions in online content. |
| (Geetha et al., 2023) | 2023 | Amazon, Yelp | Presented the 'Amazon and Yelp Reviews' dataset as a valuable resource for sentiment analysis. |
| (Dharrao et al., 2023) | 2023 | Amazon | Explored the use of NLP to analyze Amazon reviews. |
| (Kumar, 2023) | 2023 | Amazon | Employed machine learning techniques, including NLP and deep learning algorithms, to analyze Amazon product reviews. |
| (Mehul et al., 2023) | 2023 | Amazon, Yelp | Focused on sentiment polarity analysis for e-commerce customer reviews. |
| (Solairaj et al., 2023) | 2023 | Amazon | Presented an EESNN-SA-OPR method utilizing CF and product-to-product similarity. |
| (Sumathi & Santharam, 2023) | 2023 | Amazon | Investigated business strategies for customer retention and attraction, employing NLP-based sentiment analysis. |
| (Qorich & El Ouazzani, 2023) | 2023 | Amazon | Introduced a CNN model for sentiment analysis in internet reviews. |
| (Elangovan & Subedha, 2023) | 2023 | Cell Phones and Accessories dataset, Amazon | Introduced the APGWO-DLSA method to enhance sentiment analysis in online product reviews. |
| (Kaur & Sharma, 2023) | 2023 | SemEval-2014, Sentiment140, STS-Gold | Outlined a consumer review summarization model using NLP and LSTM. |
| (Venkataraman & Jadhav, 2023) | 2023 | Amazon | Investigated leveraging post-purchase customer reviews, particularly focusing on mobile phone reviews. |
| (Meghana et al., 2023) | 2023 | Amazon | Utilized sentiment analysis to comprehensively assess online product reviews. |
| (Kshirsagar et al., 2023) | 2023 | Twitter | Conducted sentiment analysis through data mining on various platforms, including Twitter. |
