# Supplementary material for: Natural language processing for analyzing online customer reviews: a survey, taxonomy, and open research challenges
Source: PeerJ Comput Sci. 2024 Jul 19;10:e2203. doi: 10.7717/peerj-cs.2203 (PMC11323031; doi:10.7717/peerj-cs.2203)
Supplement: Supplemental Information 5 [file peerj-cs-10-2203-s005.docx]

Supplemental Table S3. Summary of literature on review analysis and management.

| Ref. | Year | Dataset | Description |
| --- | --- | --- | --- |
| (Wimmer & Yoon, 2015) | 2015 | eBay, Amazon | Proposed AI framework for automated detection of counterfeit products using NLP and topic modeling. |
| (Ata Ur et al., 2019) | 2019 | Amazon, Flipkart, Daraz | Introduced an Intelligent Interface for detecting and eliminating fake product reviews on major e-commerce platforms with 87% accuracy. |
| (Lutz et al., 2019) | 2019 | Amazon | Challenged the belief that longer online reviews are universally more helpful, suggesting impact depends on argumentation within the text. |
| (Jin et al., 2019) | 2019 | Yelp | Combated machine-generated fake reviews by integrating business information and user reviews using an encoder-decoder model. |
| (Sadman et al., 2020) | 2020 | Amazon, Yelp, Google, Facebook | Addressed the challenge of detecting deceptive online reviews and ratings using a historical stylometry-based methodology. |
| (Xu et al., 2020) | 2020 | Amazon | Introduced a model combining BERT features with deep learning techniques to predict the helpfulness of online customer product reviews. |
| (Bilal et al., 2021) | 2021 | Yelp | Predicted review helpfulness using regression and classification, incorporating SNS features. |
| (Jain et al., 2021) | 2021 | Yelp | Explored the influence of online reviews on consumer decisions, proposing a supervised approach to detect opinion spammers in reviews. |
| (Anas & Kumari, 2021) | 2021 | Amazon, Yelp | Focused on using NLP techniques and machine learning models to detect and eliminate fake reviews. |
| (Birim et al., 2022) | 2022 | Amazon | Investigated optimal feature combinations for fake review detection, emphasizing the importance of behavior-related features. |
| (Aishwarya & Prashanth Kumar, 2023) | 2023 | Amazon, Yelp | Introduced a hybrid CNN-LSTM deep learning model with sentiment analysis to detect fraudulent reviews. |
| (Thilagavathy et al., 2023) | 2023 | Reviews of 20 Hotels | Utilized supervised machine learning and NLP to detect and eliminate fake reviews, focusing on counterfeit product evaluations influencing customer decisions. |
| (Iliev et al., 2023) | 2023 | Amazon | Outlined a Python-based SVM system to identify and differentiate fake product reviews. |
| (Deshai & Bhaskara Rao, 2023) | 2023 | Ott, Amazon, Yelp, TripAdvisor, IMDb | Proposed an effective method using CNNs and adaptive particle swarm optimization with NLP to detect fake online reviews with 99.4% accuracy. |
| (Bilal & Almazroi, 2023) | 2023 | Yelp | Tackled information overload in online reviews by proposing a solution using fine-tuned BERT models. |
