# Supplementary material for: Natural language processing for analyzing online customer reviews: a survey, taxonomy, and open research challenges
Source: PeerJ Comput Sci. 2024 Jul 19;10:e2203. doi: 10.7717/peerj-cs.2203 (PMC11323031; doi:10.7717/peerj-cs.2203)
Supplement: Supplemental Information 6 [file peerj-cs-10-2203-s006.docx]

Supplemental Table S4. Summary of literature on customer experience and satisfaction.

| Ref. | Year | Dataset | Description |
| --- | --- | --- | --- |
| (Marrese-Taylor et al., 2013) | 2013 | TripAdvisor | Extracted consumer preferences from tourism reviews using aspect-based opinion mining with a 35% average extraction. |
| (Tian et al., 2016) | 2016 | TripAdvisor | Analyzed English hotel reviews in Chinese cities for managerial insights using NLP, text mining, and sentiment analysis. |
| (Hu et al., 2017) | 2017 | TripAdvisor | Generated hotel summaries from travel forums, considering author credibility and conflicting opinions for improved performance. |
| (Rogojanu et al., 2018) | 2018 | - | Integrated eCommerce and offline retail using NLP and Speech-to-Text for efficient checkout and smart shop solutions. |
| (Limsopatham et al., 2018) | 2018 | Alibaba, Amazon, eBay | Leveraged automatic speech recognition to create artificial personal shoppers for eCommerce, enhancing user trust through human-like conversations. |
| (Ghani et al., 2018) | 2018 | Amazon | Measured customer loyalty using aggregated sentiment scores and fuzzy logic with 94% accuracy on Amazon.com data. |
| (John et al., 2019) | 2019 | Amazon | Used NLP to extract insights from user-generated reviews in the nutraceutical retail vertical for better decision-making. |
| (Ashfaq & Kausar, 2019) | 2019 | Amazon | Proposed a rapid customer loyalty model for e-commerce with a 72% loyalty rate from Amazon.com reviews. |
| (Ching & De Dios Bulos, 2019) | 2019 | Yelp | Predicted customer concerns for restaurants using sentiment analysis and opinion mining on Yelp datasets. |
| (Chang et al., 2019) | 2019 | TripAdvisor | Integrated sentiment analysis, aspect extraction, and visual analytics for improved hotel reviews analysis. |
| (Srivastava & Kalro, 2019) | 2019 | Yelp | Examined online review helpfulness using the Elaboration Likelihood Model, revealing the impact of latent content factors. |
| (Sann & Lai, 2020) | 2020 | TripAdvisor | Applied Aspect-Based Sentiment Analysis to categorize hotel-related service failures, highlighting cultural differences. |
| (Christodoulou et al., 2020) | 2020 | TripAdvisor | Analyzed sentiment and topics among Cyprus tourists using logistic regression and NLP. |
| (van Vliet et al., 2020) | 2020 | App Store Reviews | Investigated a crowdsourcing approach for efficiently classifying user feedback on app stores and social media. |
| (Herbst et al., 2020) | 2020 | Yelp | Examined parental preferences for child care using Yelp.com reviews, revealing income-dependent satisfaction variations. |
| (Bhatia, 2021) | 2021 | Standard Opinosis Dataset | Explored opinion summarization in Web 3.0 e-commerce platforms using abstractive and extractive techniques. |
| (Moharkar et al., 2022) | 2022 | Amazon | Enhanced review-based question answering systems using advanced NLP models like BERT and BART. |
| (Sergiacomi et al., 2022) | 2022 | TripAdvisor | Surveyed reviews of Croatia's Plitvice Lakes National Park to identify management topics, strengths, and weaknesses. |
| (Barbierato et al., 2022) | 2022 | TripAdvisor | Investigated success factors of wine tours in Tuscany using text mining and sentiment analysis. |
| (Ratmele & Thakur, 2022) | 2022 | Amazon | Introduced a hierarchical attention network-based framework for analyzing Amazon Smartphone reviews. |
| (Suresh & Gurumoorthy, 2022) | 2022 | Amazon | Used sentiment analysis to classify smartphone reviews and predict product ratings based on user feedback. |
| (Hsu et al., 2022) | 2022 | Amazon QA Corpus (COQASUM) | Introduced a novel CQA summarization task to address information overload in Community-based Question Answering platforms. |
| (Alves et al., 2022) | 2022 | Amazon | Investigated the utility of pre-trained transformers in extracting customer sentiment from online reviews. |
| (Nainwal et al., 2023) | 2023 | Amazon | Applied machine learning, NLP, and deep learning for text summarization of product reviews, reducing reading time and enhancing understanding. |
