# Supplementary material for: Natural language processing for analyzing online customer reviews: a survey, taxonomy, and open research challenges
Source: PeerJ Comput Sci. 2024 Jul 19;10:e2203. doi: 10.7717/peerj-cs.2203 (PMC11323031; doi:10.7717/peerj-cs.2203)
Supplement: Supplemental Information 7 [file peerj-cs-10-2203-s007.docx]

Supplemental Table S5. Summary of literature on user profiling and recommendation systems.

| Ref. | Year | Dataset | Description |
| --- | --- | --- | --- |
| (Wang et al., 2017) | 2017 | Yelp | Introduced VFDSR, a service recommendation algorithm using fine-grained value features from customer reviews, demonstrating superior performance. |
| (Wang et al., 2018) | 2018 | Amazon | Proposed LRMM, a framework for multimodal learning in content-based recommendation, excelling in rating prediction and handling data-sparsity. |
| (Neeraj et al., 2018) | 2018 | Amazon | Utilized data mining, psychology, and NLP to enhance recommender-based mobile apps' profitability and usability. |
| (Hüsson et al., 2020) | 2020 | - | Investigated intelligent personal assistants in business workflows, introducing an explanation mode for speech interaction in ERP software. |
| (Xia et al., 2021) | 2021 | Amazon | Introduced DAMIN, a deep learning model showing improved click-through rate prediction. |
| (Zhuang & Kim, 2021) | 2021 | TripAdvisor | Refined a BERT model for a multi-criteria hotel recommender system, outperforming single-criteria benchmarks. |
| (Tahir & Asif Naeem, 2022) | 2022 | Roman Urdu Tweets, Google Reviews | Explored user interests in the Pakistani fashion industry using LDA, LSA, BERT, sentiment analysis, and K-Means clustering. |
| (Patidar & Patel, 2022) | 2022 | Amazon | Outlined a product recommender model using NLP on customer reviews, showing notable performance gains in multi-node clusters. |
| (Lee et al., 2022) | 2022 | Rotten Tomatoes, Amazon | Introduced a graph-based movie recommender system, outperforming conventional models on Kaggle datasets. |
| (Acharya et al., 2023) | 2023 | Amazon | Explored the influence of curiosity and focused immersion on AI-driven Recommender Systems in e-commerce. |
| (Noorian et al., 2023) | 2023 | TripAdvisor, Yelp | Proposed a tourist recommendation system using Neural Network-LSTM and Bidirectional Encoder Representations from Transformer. |
| (Paul & Singh, 2023) | 2023 | Amazon | Proposed a weighted hybrid recommendation system using sentiment analysis and CF, resulting in enhanced precision. |
| (Ananth et al., 2023) | 2023 | Amazon, Flipkart | Introduced FusionSCF, a model addressing cold-start and long-tail issues in Recommendation Systems by combining CF with sentiment analysis. |
