# Supplementary material for: Natural language processing for analyzing online customer reviews: a survey, taxonomy, and open research challenges
Source: PeerJ Comput Sci. 2024 Jul 19;10:e2203. doi: 10.7717/peerj-cs.2203 (PMC11323031; doi:10.7717/peerj-cs.2203)
Supplement: Supplemental Information 8 [file peerj-cs-10-2203-s008.docx]

Supplemental Table S6. Summary of literature on marketing and brand management.

| Ref. | Year | Dataset | Description |
| --- | --- | --- | --- |
| (Cui et al., 2013) | 2013 | eBay, Amazon | Introduced a novel algorithm combining opinion mining and dependency relation analysis for accurate e-commerce feedback comments. |
| (Zhang et al., 2014) | 2014 | eBay, Amazon | Outlined CommTrust, a trust evaluation approach in e-commerce addressing the 'all good reputation' issue. |
| (Angioni et al., 2015) | 2015 | Yelp | Experimentally integrated Opinion Mining and CF, revealing user inconsistencies in star ratings alignment. |
| (Bhargava et al., 2016) | 2016 | Amazon, Flipkart | Pioneered an approach to compute seller trust in e-commerce through fine-grained analysis of user feedback comments. |
| (Kokkodis & Lappas, 2016) | 2016 | Amazon | Investigated the influence of Amazon's Verified Purchase badge on review helpfulness and product ratings. |
| (Sorokina & Cantú-Paz, 2016) | 2016 | Amazon | Detailed efforts to enhance Amazon Search's relevance ranking using diverse algorithms and NLP techniques. |
| (Anjum & Sabharwal, 2016) | 2016 | Amazon | Investigated methods for analyzing consumer opinions, proposing a hybrid approach to effectively rank products. |
| (Adams et al., 2017) | 2017 | Amazon | Focused on automating the discovery of safety and efficacy concerns in OTC joint and muscle pain relief products using "smoke word" dictionaries. |
| (Megasari et al., 2018) | 2018 | Amazon | Investigated the ability of models to gauge skill acquisition in online review writing, focusing on the evolution of this skill over a sequence of reviews. |
| (Kumar, 2018) | 2018 | Flipkart and Amazon | Introduced a method using multi-criteria decision-making to recommend the best product based on sentiment analysis. |
| (Clavel Quintero & Arco García, 2018) | 2018 | Twitter, Amazon, SemEval-2018 | Detected irony in social media and e-commerce texts using character language model classifiers. |
| (Mastan Rao et al., 2018) | 2018 | - | Addressed challenges of inaccurate user-generated reviews in E-commerce using NLP techniques. |
| (Panigrahi & Asha, 2018) | 2018 | TripAdvisor | Presented Ranking Hotels using Aspect Level Sentiment Analysis (RHALSA) algorithm. |
| (Kauffmann et al., 2019) | 2019 | Amazon | Outlined a methodological framework utilizing sentiment analysis and NLP techniques to analyze online reviews. |
| (Galli et al., 2019) | 2019 | Yelp | Examined the influence of technology, proposing a methodology to analyze reviews for assessing business attractiveness. |
| (Koneru et al., 2019) | 2019 | Amazon | Introduced a Feature-Based Product Recommendation system using NLP and sentiment analysis. |
| (Li et al., 2020) | 2020 | Amazon | Introduced the Level of Success model (LOS) for assessing product market impact in the evolving e-commerce landscape. |
| (Mitra & Jenamani, 2020) | 2020 | Amazon | Proposed a model to quantify Online Brand Image (OBIM) by analyzing consumer reviews. |
| (Yang et al., 2020) | 2020 | Amazon | Employed Python, AHP, and ARIMA/OLS models to analyze Amazon "Sunshine" product sales. |
| (Konjengbam et al., 2020) | 2020 | Amazon | Introduced an innovative Tagging Product Review (TPR) system that employed an unsupervised approach. |
| (Benlahbib & Nfaoui, 2020b) | 2020 | Amazon | Detailed the development of AmazonRep, a reputation system for Amazon customers. |
| (Benlahbib & Nfaoui, 2020a) | 2020 | IMDb, TripAdvisor, Amazon | Introduced a holistic approach to reputation generation from customer reviews. |
| (El-Dehaibi et al., 2021) | 2021 | Amazon | Investigated differentiating e-commerce products based on customer perceptions of sustainability. |
| (Liu, 2021) | 2021 | Amazon | Examined online sales strategies for various products using sentiment analysis. |
| (Nellutla et al., 2021) | 2021 | Amazon | Utilized natural processing methods to assess the impact of online product reviews on third-party sellers. |
| (Boumhidi et al., 2021) | 2021 | IMDb dataset. | Employed Bi-LSTM, RNN and NLP techniques to compute reputation scores for companies. |
| (Christodoulou et al., 2021) | 2021 | TripAdvisor | Utilized NLP and classification techniques to investigate the impact of weather conditions. |
| (Boumhidi & Nfaoui, 2021) | 2021 | IMDb, Amazon, TripAdvisor, Yelp | Outlined a novel reputation generation system for Twitter. |
| (Asseo & Niv, 2022) | 2022 | Amazon, iHerb | Examined sweetness in online food product reviews. |
| (Lutz et al., 2022) | 2022 | Amazon | Challenged the assumption that longer product reviews are universally more helpful. |
| (Honig et al., 2022) | 2022 | Amazon | Investigated domestic robot failures using reviews. |
| (Sánchez, 2022) | 2022 | TripAdvisor | Investigated the Memorable Tourist Experience (MTE) concept through reviews. |
| (Arora et al., 2022) | 2022 | Amazon | Examined sentiment analysis of customer reviews using SVM, LSTM, and CNN techniques. |
| (Tang & Guo, 2022) | 2022 | Amazon | Employed NLP-AHP for analyzing online shopping reviews, providing actionable insights. |
| (Hossain et al., 2022) | 2022 | - | Analyzed demand for smartphones in the market through social media data using NLP and machine learning models. |
| (Ravichandran & Deng, 2023) | 2023 | TripAdvisor | Developed a model integrating justice theory and service recovery literature for online customer complaints. |
| (Mantilla-Saltos et al., 2023) | 2023 | Amazon | Investigated user satisfaction with physical activity trackers using sentiment analysis. |
| (Christodoulou & Gregoriades, 2023) | 2023 | TripAdvisor | Employed text classification and topic modeling to discern consumer personalities. |
| (Ullah et al., 2023) | 2023 | Amazon | Introduced QLeBERT, a model leveraging a quality-related lexicon to predict product quality. |
| (Harth et al., 2023) | 2023 | Amazon | Outlined an algorithm leveraging language-transformer technologies to automate product requirement generation. |
| (Vollero et al., 2023) | 2023 | Amazon, Facebook | Explored the impact of the 'Amazon effect' on consumer perceptions of service attributes in Italian consumer electronics retailers. |
| (Al-Ghuribi et al., 2023) | 2023 | Amazon, Yelp | Introduced CF methods leveraging sentiment analysis on user reviews. |
| (Li et al., 2023) | 2023 | Amazon | Introduced a novel TADO model for review-based recommender systems. |
| (Harsha et al., 2023) | 2023 | Amazon | Utilized machine learning to categorize product reviews, eliminating redundancy and preprocessing text with NLP tools to train a model capable of predicting sentiment. |
